# Anti-HSP90 autoantibodies in sera of infertile women identify a dominant, conserved epitope EP6 (380-389) of HSP90 beta protein

**DOI:** 10.1186/1477-7827-9-16

**Published:** 2011-01-27

**Authors:** Eusebio S Pires, Asmita K Choudhury, Susan Idicula-Thomas, Vrinda V Khole

**Affiliations:** 1 Department of Gamete Immuno Biology, National Institute for Research in Reproductive Health (ICMR), JM Street, Parel, Mumbai 400012, India; 2 Biomedical Informatics Center of ICMR, National Institute for Research in Reproductive Health, JM Street, Parel, Mumbai 400012, India; 3 Department of Cell Biology, School of Medicine, University of Virginia, Charlottesville 22903, Virginia, USA

## Abstract

**Background:**

We earlier reported a simple specific test for detection of anti-ovarian antibodies in infertile women and identified number of specific molecular and cellular targets of which human heat shock protein 90-beta (HSP90 beta) was found to be the most immunodominant. The present study focuses on prediction and validation of the immunodominant epitope/s of this protein using sera from infertile women having anti-HSP90 autoantibodies.

**Methods:**

Delineation of the immunodominant epitopes of HSP90 beta was done by using epitope prediction algorithms and 10 peptides (EP1-EP10) were custom synthesized. Their immunoreactivity was measured by ELISA using sera from patients and controls. To determine the most immunodominant epitope, the results were subjected to statistical analysis. The immunoreactivity of the immunodominant peptides were confirmed by dot blots using sera from patients. A rabbit polyclonal antibody against the immunodominant epitope was generated and its immunoreactivity to the parent protein in ovarian extracts as well in oocytes and embryos was investigated.

**Results:**

Experimentally and statistically, peptide EP6 (380-389) seems to be the major antigenic epitope for the serum antibody binding followed by EP1 (1-12) and EP8 (488-498). Predicted 3D structures of these peptides demonstrated that they exist in the loop conformation which is the most mobile part of the protein. Also, analysis of the sequences of HSP90 beta across several species reveals that EP6 peptide forms a part of a well conserved motif. The polyclonal antibody generated to the immunodominant epitope- EP6 confirms similar biochemical and cellular immunoreactivity as seen with the patients' sera having anti-HSP90 autoantibodies.

**Conclusions:**

The decapeptide EP6 is a major immunogenic epitope of HSP90 followed by EP1 and EP8. Knowledge of binding epitopes on the autoantigen is necessary to understand the subsequent pathologic events. The study might generate new tools for the detection of disease-inducing epitopes and a possible therapeutic intervention.

## Background

Autoimmune diseases remain among the most poorly understood and recognized categories of illnesses in the world [1]. For the last few decades it has been well established that the human ovary also undergoes an autoimmune attack, which is characterized by the development of an anomalous immune response against various compartments of the organ [2,3]. Ovarian autoimmunity is known to be manifested by individuals having polycystic ovarian syndrome (PCOS), endometriosis and primary ovarian insufficiency (POI) or premature ovarian failures (POF) [2,4]. Women enrolled in the in vitro fertilization -- embryo transfer (IVF-ET) program have also been shown to have AOA and these have been correlated with poor reproductive outcomes [5]. Though various causes such as chromosomal, enzymatic, iatrogenic etc are known to be involved in pathophysiological condition of POI, about 1% of the total cases are known to be caused due to autoimmunity [6]. These women exhibit a hypergonadotropic - hypoestrogenic hormone profile and antiovarian antibodies (AOA) are detected in the sera of these women [2,4]. However, the involvement of other autoimmune disorders along with POI cannot be completely ruled out as various Systemic Lupus Erythromatosus, Graves's disease, Addison's disease also manifest POI [7].

Detection of these AOA has always remained a challenge for investigators as several published tests showed presence of these AOA even in controls [8]. Until recently there was no validated serum marker/s that could establish a diagnosis of ovarian insufficiency with certainty [9]. The specificity of the existing AOA tests has been questioned because of its false positive results. Our group has been successful in establishing a simple and specific diagnostic test to detect AOA in women with infertility [10]. We were able to demonstrate true AOA status in these women using our novel blocking approach and were able to identify several new molecular and cellular targets [11]. It was observed that the target antigens range between 30-150 kDa, of which a 90 kDa protein was shown to be the immunodominant antigen. High throughput proteomic analysis (LC/MS and MS/MS) revealed the identity of the protein to be human heat shock protein 90 beta (HSP90β) [12]. Involvement of anti-HSP90 antibodies in pathogenesis of several diseases such as systemic lupus erythematosus [13], rheumatoid arthritis [14], osteocarcinoma [15] and ovarian cancer [16] has been reported. However, we were the first ones to demonstrate the presence of anti-HSP90β antibodies in women with infertility [12].

Having established this, we embarked upon identification of the immunodominant epitopes of HSP90β, knowing the fact that the whole protein by itself is unlikely to be antigenic. We did this by using epitope prediction algorithms and then confirmed them by wet-lab experiments using the sera from infertile women having autoantibody to HSP90 and normal fertile individuals who served as controls. Epitoimmunomics is a relatively upcoming branch of modern day biology and epitopic peptides are long known as highly versatile molecules for a variety of biological and immunological applications. Unlike proteins which unfold readily and subsequently loose their activities or in which the active antigenic components lie buried in its 3D structure making it inaccessible to the immune system; peptides are preferred choice as they are functionally quite stable, making them suitable molecules for facile and robust screening assays [17]. Synthetic peptides mimicking epitopes, as well as anti-peptide antibodies, have many applications in the diagnosis of various human diseases [18,19]. Also, attempts have been made to develop peptide based synthetic prophylactic vaccines for various infections, as well as therapeutic vaccines for chronic infections and noninfectious diseases, including autoimmune diseases, neurological disorders, allergies, and cancers [20]. The majority of available epitope prediction methods focus on continuous epitopes due to the convenience of the investigation in which the amino acid sequence of a protein is taken as the input. Such in silico prediction methods are based upon the amino acid properties including hydrophilicity [21,22], solvent accessibility [23], secondary structure [24], flexibility [25] and antigenicity [26].

In the present study we predicted, identified, characterized and validated the antibody binding regions of HSP90β using (a) Bioinformatics and (b) sera from infertile women having these auto-antibodies. Identification of epitopic peptides targeted by autoantibodies will facilitate development of sensitive assays for diagnosis of autoimmune diseases. Also, important insights into the pathophysiology of autoimmune disorders can be gathered by characterizing the epitopes targeted by these antibodies.

## Methods

### Study groups

The study was approved by the Institute's clinical ethics committee and informed written consent was obtained from the participants. Briefly, women with POI and infertile women enrolled for IVF-ET constituted the study groups (discussed in detail in [12]). Referring physicians made the diagnosis of POI based on the following criteria: onset of amenorrhea (for at least 4-6 months) before attaining the age of 40 years and high serum follicle-stimulating hormone (FSH) levels > 40 IU/liter (estimated twice at least 1 month apart). Women who developed POI as a result of surgery or radiation/chemotherapy, those having a genetic involvement, abnormal karyotypes, infections/viral oophoritis, or galactosemia-induced POI were excluded from the study. The inclusion criteria for patient selection in the IVF-ET program were those women who were less than 40 years of age at the time of enrollment in the study presenting with infertility and for whom IVF-ET had been determined to be the next treatment for conception. These women had a normal baseline pelvic ultrasound examination and a normal uterine cavity. Patients with the following conditions were excluded from the study: those who had pelvic surgery within the past 8 weeks, including appendicitis or uterine or ovarian surgery, excluding diagnostic laparoscopy; those who had clinical evidence of pelvic inflammatory disease within the past 3 months and who were treated for any viral infection; endometriosis; women who had submucous or intramural fibroids of any size and women who had hydrosalpinx and had polycystic ovary syndrome. None of the patients' had been treated for any form of autoimmune diseases such as systemic lupus erythematosus, thyroid or adrenal autoimmunity and diabetes mellitus. Sera were collected from 64 women having anti-HSP90 antibodies and 40 proven fertile healthy women (22-34 years old). All these control women were parents and had no previous history of irregular menses nor were treated for any autoimmune disease. None of the controls showed the presence of AOA.

### Animals

Inbred Belgium white adult rabbits and Swiss mice were housed in a temperature-controlled room with a 12-hr light cycle. All animal care practices and experimental procedures complied with the guidelines of the Care and Prevention Society against Cruelty of Experimental Animals (CPSCEA) on animal care and were approved by the Institutional Animal Ethics Committee.

### Western blotting

In brief, 10 μg of commercially available human ovarian extract (Clontech, USA) / 40 μg mice ovarian extract were separated on a 10% gel by electrophoresis at 80 V for 3 h [27] and then transferred to nitrocellulose membranes (GE Healthcare, USA) using an electro blotting apparatus (BioRad Labs, Richmond, CA, USA) at 80 V for 2 h [28]. The individual lanes were blocked for 1 h at room temperature with 5 g% non-fat dry milk (Nakoda, India) in phosphate buffered saline (NFDM-PBS). The blots were incubated overnight at 4°C with polyclonal antibodies to HSP90α and HSP90β (Millipore, USA) diluted 1:200 in PBS. Monoclonal antibody to GAPDH (Calbiochem, USA) was used at a dilution of 1 in 6000 in PBS and served as a loading control to the same blots. Following day, the membrane strips were washed with 0.1% (v/v) Tween-20 (Sigma, USA) in PBS and incubated with swine anti rabbit HRP and rabbit anti mouse HRP diluted in 1% NFDM-PBS and incubated at room temperature for 1 h. Strips were then washed as described above. Detection was done using enhanced chemiluminescence plus (ECL) kit (GE Healthcare, USA) as per the standard protocol.

### Prediction of antigenic peptides using *in silico *tools

The antibody epitope prediction tool available at IEDB analysis resource [29] was used to identify the putative epitopes of the protein. Two algorithms i.e. Kolaskar and Tongaonkar antigenicity [26] and Bepipred linear epitope prediction [30] methods were selected for this purpose. Ten putative epitopes were short listed based on the following criteria: a) They should either have an overlap of predicted antigenic regions as per the 2 algorithms or in cases where the predicted sites are adjacent to each other the epitopes selected were in the regions harboring predicted sites as per the above two algorithms; b) The physicochemical parameters (viz. hydrophobicity, flexibility, surface accessibility) are suggestive of a putative epitope [31]. In order to avoid nonspecific antibody reactions, the 10 putative epitopes were checked for sequence similarities with proteins deposited in the NCBI database. The online BLASTP tool available at NCBI [32] was used for the database search of these peptides. Multiple sequence alignment was carried out using CLUSTALW2 program of EBI [33] to understand the conservation of the predicted epitopes of the protein across various species for which the sequence information is available at NCBI. PyMOL [34] was used for visualization of the 3D structures of the predicted epitopes and rendering the molecular images.

### Peptide synthesis

Based on the epitope prediction algorithm, 10 peptides (labeled as EP1 to EP10) were shortlisted and were custom synthesized by solid-phase synthesis using Fmoc chemistry. These analytical grade peptides (99% purity) were then conjugated to keyhole limpet hemocyanin (KLH) linker. Synthesis and conjugation of the 10 peptides was done at a commercial facility of US Vitamins Ltd., Mumbai.

### Enzyme linked immunosorbant assay (ELISA)

Ninety six well microtitre plate (Nunc, Denmark) were coated with the 10 KLH-conjugated HSP90β peptides in individual wells at a concentration of 1 μg using carbonate- bicarbonate coating buffer, pH 9.6, by incubating at 4°C overnight. Excess solution was flicked off the next day and modules were washed with PBS containing 0.05% (v/v) Tween-20 (Sigma, USA). Nonspecific binding sites were blocked with 5 g% NFDM in PBS, for 1 hr at room temperature. Patient and control sera in duplicates were added to the wells and incubated at 4°C overnight. A 'no primary antibody' served as negative control where sera were replaced by equal volume of PBS. Unbound antibodies were removed off by washing wells 4 times with PBS containing 0.05% (v/v) Tween-20 (Sigma, USA). Suitably diluted (1: 1000) goat anti human HRP labeled secondary antibody (Sigma, USA) in 1 g% (w/v) NFDM in PBS was added and incubated at 37°C for 1h. The wells were then washed as described above and the immunoreactivity was visualized using o-phenylene diamine / H_2_O_2 _(Sigma, USA) prepared in substrate buffer. Reaction was stopped by adding 4N H_2_SO_4 _(Qualigens, India) and optical density was measured at 492 nm on a titreteck multiscan plate reader (Biotek, USA).

### Calculations and statistical analysis

The immunodominant epitope was determined by calculating the % change of the absorbance values for each of the peptides using the calculation: % change in absorbance = [(mean absorbance of patient -- mean absorbance of control) / mean absorbance in control] x 100. A higher increase in the percent change in absorbance value corresponds to higher antigenicity of the peptide. The cut off range for positivity towards a peptide was set as per the statistics note described by Altman and Bland [35] and Jeffoate [36]. Statistical analysis was performed using the SPSS software (Chicago, IL) version 11. The results generated from the ELISA data showed a skewed representation and for this reason we preferably selected to quote the two outer centiles such as the 80^th ^centile of controls and 20^th ^centile of patients to represent the data. We ranked the absorbance values from the controls in order of the increasing absorbance readings. The kth centile (q) is obtained using the calculation q = k (n + 1) / 100; where k -- the desired centile and n- the total number of samples screened.

### Dot blot analysis

1 μg of individual un-conjugated peptides were dotted on nitrocellulose membrane and allowed to adhere for at least 2 h at 37°C before blocking for 1 h at room temperature in 5 g% NFDM-PBS. Patient sera testing positive in ELISA or control sera diluted five fold were added to the strip and incubated at 4°C overnight. Dotted membrane with no primary antibody served as 'negative control'. Following day the membranes were washed with 0.1% (v/v) Tween-20 in PBS and then incubated with suitably diluted (1: 100,000) goat anti human HRP labeled secondary antibody (SIGMA, USA) in 1 g% (w/v) NFDM-PBS for 1 h at room temperature. Membranes were then washed as described above. Detection was done using enhanced chemiluminescence plus (ECL) kit (GE Healthcare, USA) as per the standard protocol.

### Production of polyclonal antibody to immunodominant peptide EP6

Prior to immunization sufficient pre immune blood was collected through the marginal ear vein of the rabbit. The serum was separated and stored at -20°C till further use. Immunization protocol was carried out as described earlier [10] with slight modifications.

Briefly, 100 μg of peptide EP6 conjugated to KLH was dissolved in 1ml of 0.01 M PBS pH 7.4, and mixed with equal volume of Freund's complete adjuvant (SIGMA, USA) and injected subcutaneously at approximately 30 sites. Two boosters at an interval of 15 days were given using Freund's incomplete adjuvant (SIGMA, USA) and post immune serum was collected 10 days after the last booster, stored as aliquots at --20°C. This post immune serum containing the anti- EP6 peptide antibody was used for subsequent experiments.

### Immunochemical characterization of the polyclonal antibody to peptide EP6

#### Dot blot analysis

The immunoreactivity of the rabbit sera post immunization with the dominant EP6 peptide was checked for its specificity by dot blot at a dilution of 1:50 using the same protocol with appropriate reagents as described earlier in this section.

#### Western blot analysis

In brief, 160 ng of recombinant human HSP90 protein (rhHSP90) (Calbiochem, USA) and 40 μg of mice ovarian protein were separated on a 10% gel by electrophoresis as described earlier in this section. The individual lanes were blocked for 1 h at room temperature with 5g% (w/v) NFDM-PBS. Pre-immune (serum of animal prior to injecting the peptide, serves as a true negative control) and post-immune rabbit sera diluted fifty fold with blocking buffer were added to the strip and incubated at 4°C overnight. Following day the membrane strips were washed with 0.1% (v/v) Tween-20 in PBS and then incubated with suitably diluted (1: 3,000) swine anti rabbit HRP labeled secondary antibody (Dako, Germany) in 1 g% (w/v) NFDM-PBS for 1 h at room temperature. Strips were then washed as described above. Detection was done using enhanced chemiluminescence plus (ECL) kit (GE Healthcare, USA) as per the standard protocol.

#### Indirect immunofluorescence (IIF)

Adult Swiss female mice were injected intraperitonealy with 10 IU pregnant mare serumgonadotropin (PMSG) (Sigma, USA) to stimulate follicular development and 48 h later with 10 IU human chorionic gonadotropin (hCG) (Bharat Serum, India) to induce ovulation. 16 to 18 h after hCG injection, mice were euthanized by cervical dislocation and oocytes were harvested from the oviducts. For collection of embryos, females were bred singly to proven fertile mature male mice immediately after the hCG injection and checked for a vaginal plug the following morning. Embryos were flushed from the oviducts of plug positive females at different time points for 2-cell, 4-cell, 8-cell and morula. The oocytes / embryos were fixed in 4% (w/v) para-formaldehyde for 15 min at 4°C. Further, these were permeabilized with 0.1% Triton-X 100 in PBS for 10 minutes. Blocking was carried out using 5% (v/v) normal swine serum (NSS) (Dako, Germany) made in 5 g% (w/v) Bovine Serum Albumin (Sigma, USA) in PBS for 1 hr at room temperature. The oocytes and embryos were incubated with pre-immune and post-immune sera diluted fifty fold in blocking buffer overnight at 4°C in a moist chamber. Next day, three washes in PBS for 5 min each were given and a secondary antibody swine anti rabbit FITC conjugate diluted two hundred fold in PBS (Dako, Germany) was added onto the slides and incubated for 1 hr at room temperature. Cells were counterstained with 0.1% (w/v) DAPI (Roche, USA) which was added along with secondary antibody. Excess antibody and dye were removed by washing thrice with PBS for 5 min each. Slides were mounted on prolong gold antifade (Molecular Probes, USA) and immunostaining was observed under Confocal microscope at 640X magnification (Carl Zeiss, Germany).

## Results

### HSP90β is the predominant isoform present in ovarian extracts

**Figure 1:**
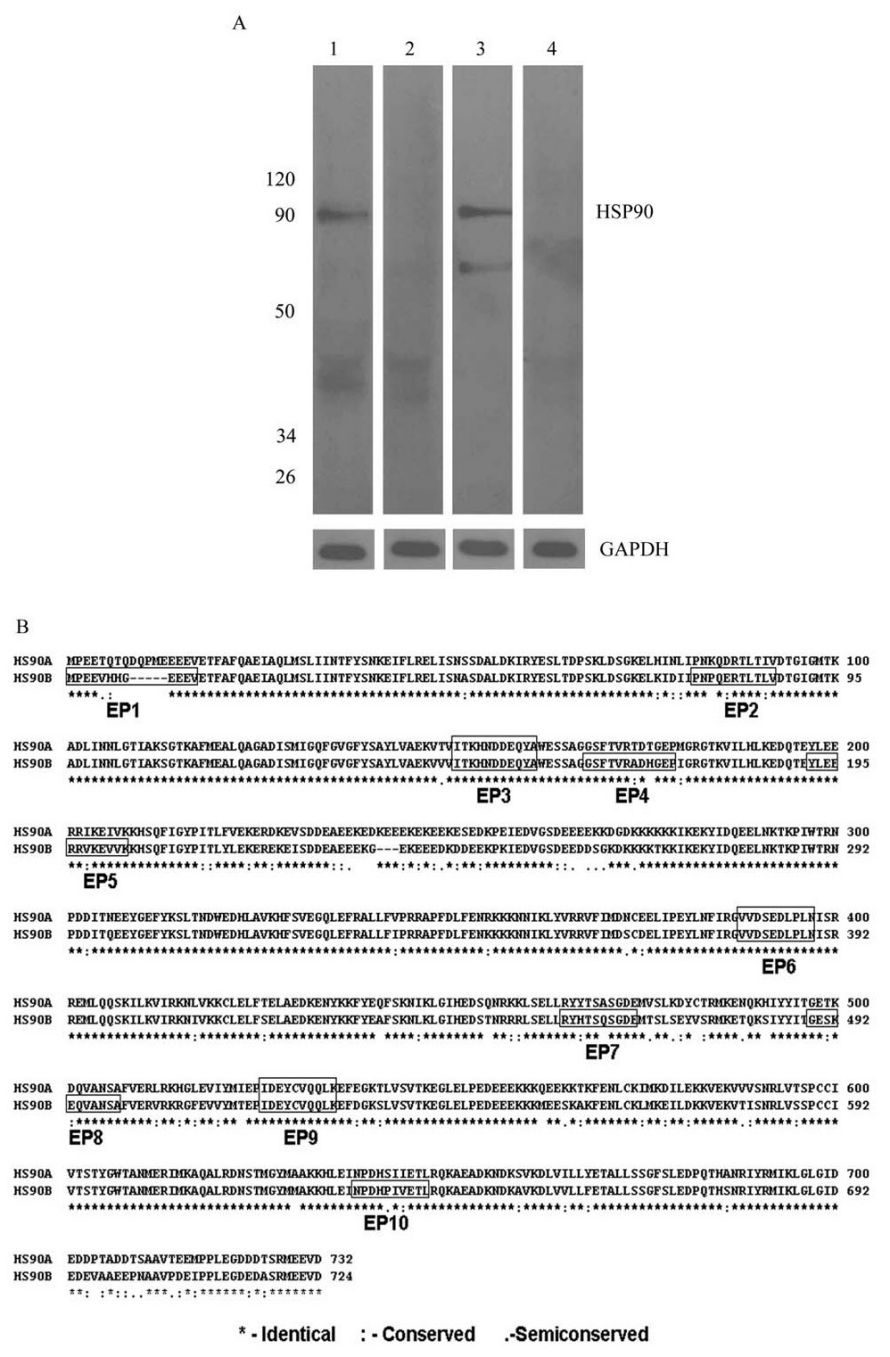
**Putative epitopes of HSP90β, an ovarian specific isoform**. A) Western blot analysis depicting the major isoform in total human ovarian extracts to be beta isoform as seen at the 90 kDa loci when probed with HSP90β antibody (lane 1). No immunoreactivity was seen with HSP90α antibody (lane 2). Mouse testicular extracts were used as positive control for the HSP90α antibody (lane 3). 'No primary/ secondary alone' antibodies served as negative control (lane 4). Antibody to GAPDH served as loading control to ensure equal amounts of protein per lane. B) The figure is a ClustalW2 alignment of the alpha and beta isoform of HSP90 and it maps the 10 short listed peptides (EP1-EP10) indicating its position relative to the isoforms.

Our earlier LC-MS/ MS-MS observation revealed HSP90β to be the major immunodominant target involved in ovarian autoimmunity [12] which is also supported by the Western blot as shown in Figure 1A, which clearly demonstrates the predominance of the beta isoform of HSP90 in human ovarian protein extracts (lane 1). No immunoreactivity to the 90 kDa locus was seen when the strip was probed with HSP90α polyclonal antibody (lane 2), thereby suggesting HSP90β to be the predominant isoform in human ovary. Mouse testes extract was used as a positive control for HSP90α (lane 3). A 'no primary' control showed no immunoreactivity to any of the ovarian proteins (lane 4). GAPDH served as an equal loading control.

### Putative epitopes of human HSP90β were predicted using *in silico *methods

*In silico*studies were conducted to predict the antigenic peptides of HSP90β and 10 putative epitopes, namely EP1-EP10 were selected. Figure 1B is a ClustualW2 alignment mapping of the 10 predicted peptides (EP1- EP10). The start and end position of the peptides has been marked in this figure. BLASTP algorithm of all the 10 peptides revealed that they occur with 100% identity only in the heat shock proteins of 90 kDa family and hence there was no possibility of getting nonspecific antibody reactions with these peptides.

### Peptide immunoreactivity was validated with HSP90 positive AOA sera

#### Peptide immunoreactivity was measured by ELISA

**Figure 2:**
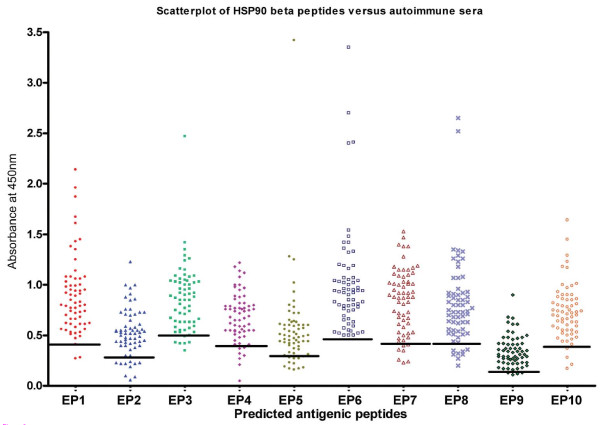
**EP6 is an immunodominant peptide of HSP90β**. Epitopic peptides were selected by the epitope prediction algorithm by Kolaskar-Tongaonkar and Bepipred. These peptides were then custom synthesized using Fmoc chemistry and used in ELISA. The figure depicts the immunoreactivity of sera from infertile women with the 10 peptides. The solid horizontal bar indicates the 80^th ^centile of controls. Values above the bar indicate a positive immunoreactivity to that peptide and values below or on the bar depicts poor or no immunoreactivity. Peptide EP6 shows strong immunoreactivity with all the 64 sera tested in ELISA with no values falling below or on the bar. All outliers i.e. sera showing absorbance values of 2 and above were not used in statistical analysis and calculations to avoid ambiguity of results.

The immunoreactivity of the sera from controls and from women having HSP90 autoantibody (patients n = 64 and controls n = 40) were tested and measured against all the 10 peptides by ELISA. The values obtained have been plotted on the scatter plot as shown in Figure 2. The increase in % change of the mean absorbance between patients and controls was calculated for each of the peptides. Also, the baseline cut off range for each of the peptide was calculated using 80^th ^centile of the control in the centile distribution statistical analysis.

The immunoreactivity of all the sera against the peptide EP6 was found to be above the baseline readings from controls. In addition to this it was seen that all 64 antibody positive patient's sera showed significant immunoreactivity to EP6 peptide. Also, as shown in Table 1, it is apparent that of all the 10 peptides EP6 demonstrated the highest increase in % change in absorbance yielding a + 69% increase in score. The increase in positive % change in absorbance designates the peptide to be more immunogenic than the others. Following EP6, EP1 demonstrated a + 54% and EP8 demonstrated a + 17% increase change. EP10 and EP3 showed a + 8% increase change. EP2, EP4 and EP7 showed no % increase change, peptides EP5 and EP9 demonstrated a decrease in % change values. In view of our statistical analysis, our data clearly indicates that epitope EP6 seems to be the major antigen binding region for the patients' serum having HSP90 autoantibodies followed by peptides EP1 and EP8.

**Table 1:** Derivation of the most immunodominant epitope of HSP90 beta to serve as a diagnostic marker

S.NO	PEPTIDES	SEQUENCE	MEAN ABSORBANCE	MEAN ABSORBANCE	CHANGE IN ABS	% INCREASE / DECREASE	RANK AS MARKER
			PATIENT ± SD	CONTROL ± SD	[**P -C / C X 100**]	CHANGE IN ABSORBANCE	
**1**	**EP1**	**MPEEVHHGEEEV**	**0.89 ± 0.39**	**0.35 ± 0.12**	**154.2**	**54%**	**2**
2	EP2	IPNPQERTLTLV	0.52 ± 0.24	0.26 ± 0.1	100	0.00%	6
3	EP3	ITKHNDDEQYA	0.83 ± 0.26	0.4 ± 0.15	107.5	8%	5
4	EP4	GSFTVRADHGEP	0.68 ± 0.24	0.34 ± 0.09	100	0%	6
5	EP5	YLEERRVKEVVK	0.51 ± 0.23	0.29 ± 0.08	75.86	-24.14%	8
**6**	**EP6**	**VVDSEDLPLN**	**0.94 ± 0.33**	**0.35 ± 0.12**	**168.5**	**69%**	**1**
7	EP7	RYHTSQSGDE	0.8 ± 0.32	0.4 ± 0.12	100	0.00%	6
**8**	**EP8**	**GESKEQVANSA**	**0.78 ± 0.29**	**0.36 ± 0.09**	**116.6**	**17%**	**3**
9	EP9	IDEYCVQQLK	0.34 ± 0.15	0.19 ± 0.06	78.94	-21%	7
10	EP10	NPDHPIVETL	0.73 ± 0.27	0.35 ± 0.14	108.5	8.50%	4

Higher the value in the % increase in change of the absorbance, greater is the indication of the peptide to be the immunodominant epitope for that protein. Peptides giving a negative value (decrease change or no change) are not good candidates to be used as markers. Of all the 10 peptides, EP6 ranked first and showed the highest increase in % change in absorbance followed by EP1 and EP8.

#### Validation of the peptide immunoreactivity by dot blot

**Figure 3:**
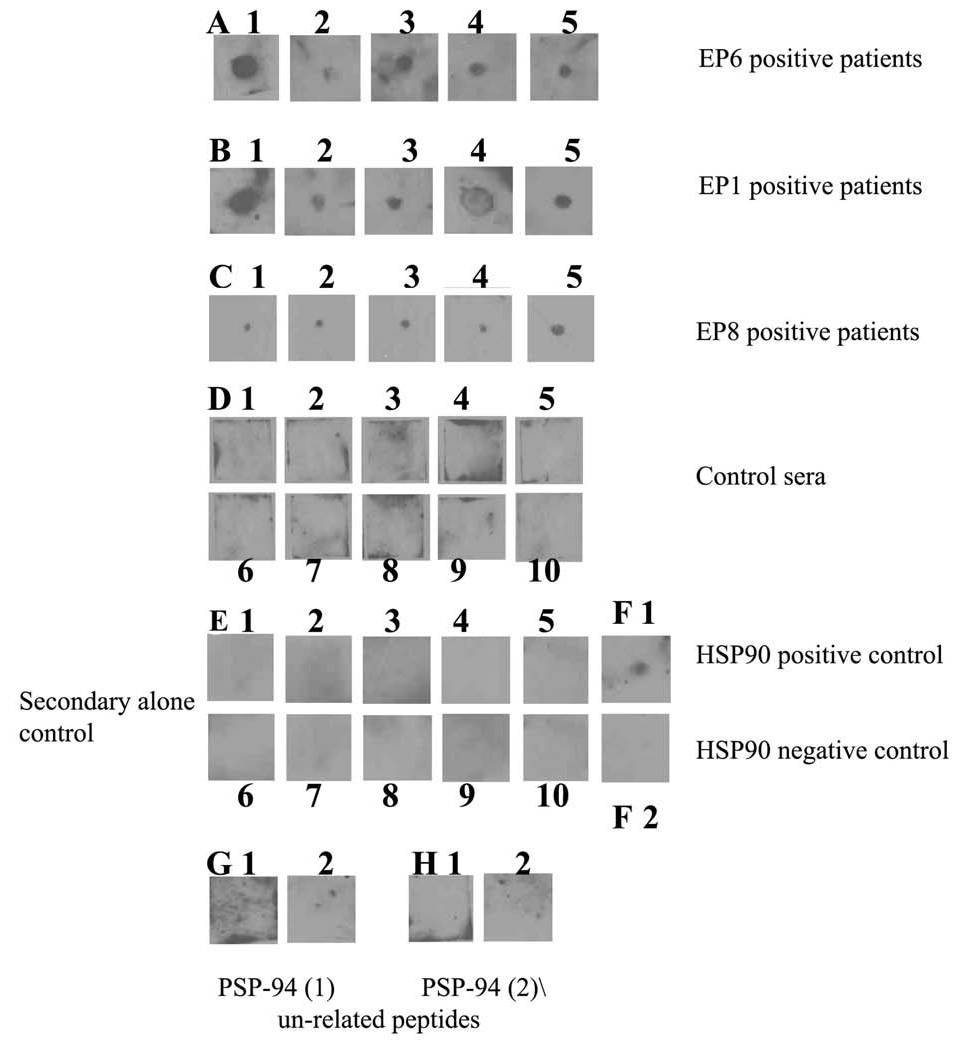
**Dot blot analysis of patient's sera with immunodominant epitopes of HSP90β**. Panels (A, B and C) are representative panels showing immunoreactivity of patients' sera testing positive in ELISA with EP6, EP1 and EP8 peptides respectively. Panel (D) shows no immunoreactivity to any of the 10 peptides probed with sera from normal fertile control serum. Panel (E1-10) demonstrates no reactivity of secondary antibody with any of the 10 peptides. Panel F1: Immunoreactivity of monoclonal to HSP90 with recombinant HSP90, serving as positive control for the assay. Panel F2: negative control for the monoclonal to HSP90. Panel (G and H): Immunoreactivity to 2 un-related peptides of Prostate Secretory Protein 94 - PSP94 (Panel G: KDPKKTCSVSE and panel H: KKEDCKYIVVEK) using both patient (G1, H1) and control (G2, H2) sera respectively.

Findings from dot blot analysis have been represented in Figure 3 panels (A, B and C) depict the immunoreactivity of patient's sera testing positive in ELISA with EP6, EP1 and EP8 dotted peptides respectively. Of all the 10 peptides screened by us, these 3 peptides were found to be the most immunodominant targets by the patient's sera. Panel (D) shows no immunoreactivity to any of the 10 peptides when the dot blot was probed with sera from normal fertile controls. Panel (E) demonstrates no involvement of secondary antibody towards any of the 10 peptides. The same panel also shows recombinant HSP90 protein reactivity with monoclonal to it, serving as positive control (Panel F1). SP2/0 myeloma culture supernatant was used as negative control for panel F1 (Panel F2). Panel (G and H) shows no immunoreactivity to 2 unrelated peptides of Prostate Secreted Protein 94 - PSP94 using both patient and control sera respectively, thereby confirming the specificity of the autoantibodies to HSP90β peptides.

### Immunochemical characterization of rabbit polyclonal antibodies to the immunodominant peptide EP6

Having established the fact that EP6 is the immunodominant peptide target by the patient's sera we characterized this antigen to confirm its similarity with the native HSP90β protein. For this we raised a polyclonal antibody which was used for characterization by dot blot, Western blot analysis and indirect immunofluorescence.

**Figure 4:**
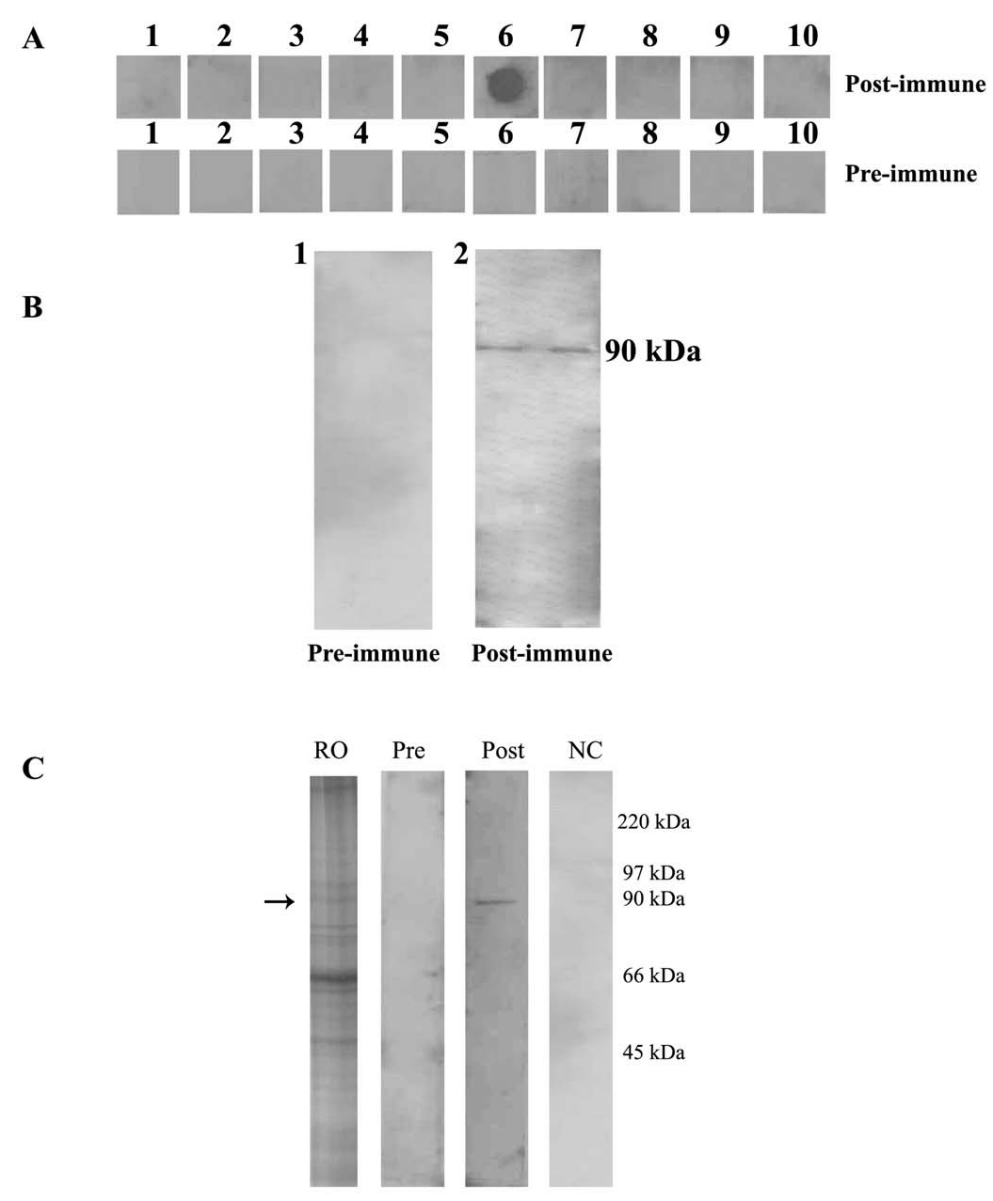
**Biochemical characterization of epitope EP6**. Panel (A) depicts dot blot analysis using rabbit polyclonal antibodies to peptides EP6. EP6 in panel A6 shows strong immunoreactivity with the post-immune sera. The pre-immune of the same rabbit did not react to any of the 10 peptides EP1 to EP10 in sequential order. Panel (B): Western blot analysis using rabbit polyclonal antibodies to peptide EP6 with recombinant HSP90 loaded in duplicates. B1: Pre immune serum shows no immunoreactivity. B2: Post immune serum reacts with the recombinant protein. Panel (C): Western blot analysis using the anti-EP6 peptide polyclonal antibodies with crude mice ovarian protein. Lane RO: total mice ovarian extract stained with Coomassie blue stain with an arrow indicating the locus representing the HSP90 protein. Lane Pre: The pre immune serum shows no immunoreactivity. Lane Post: The post-immune sera shows single band reactivity in the lane marked at the 90 kDa. Lane NC: Secondary alone control shows no reactivity.

Figure 4 (Panel A) shows the immunoreactivity seen with the post-immune rabbit sera against the 10 peptides in a dot blot analysis. The polyclonal antibody reacts specifically to peptide EP6 (dot blot marked 6). The pre-immune serum of the same rabbit did not show any reactivity to any of the 10 peptides (EP1 to EP10 labeled as 1 to 10 in order) thus demonstrating the specificity of anti peptide EP6 antibody.

It is very essential that the anti-peptide antibodies produced and raised to the peptide not only reacts to the peptide with which it was immunized but also shows immunoreactivity to the native protein. In Figure 4, a specific immunostaining at the 90 kDa region constituting the region of rhHSP90 protein was seen when the blot was probed with anti-EP6 peptide antibody (Panel B2) while preimmune rabbit serum showed no immunoreactivity (Panel B1). The post-immune sera also identified a single band at the 90 kDa region using a total ovarian extract in Western blotting (Panel C, lane Post) thereby confirming that the anti-peptide antibody not only reacts to the peptide with which it is immunized but also recognizes the recombinant HSP90 protein and the native molecule in the mice ovarian extract. The pre-immune serum (lane Pre) did not show any immunoreactivity.

**Figure 5:**
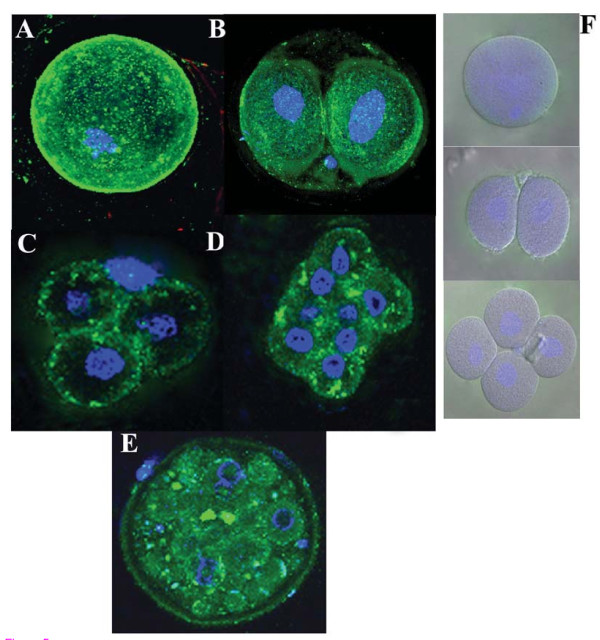
**Cellular characterization of epitope EP6**. Indirect immunofluorescence studies using the anti-EP6 peptide polyclonal antibodies. Immunoreactivity (green stain) as seen in panel (A) ovulated oocyte, (B) 2 cell embryo, (C) 4 cell embryo, (D) 8 cell embryo and (E) morula. The pre-immune of the same animal showed no immunostaining to any of the cell types as represented in bright field merged panel indicating the nucleus and cell boundary (F). DAPI (blue stain) was used to counterstain the nucleus. Images were taken on Carl Zeiss Confocal microscope at X640 magnification.

Using the anti-EP6 peptide polyclonal antibody we could see the localization in the ooplasm of the oocyte, cytoplasm of 2 cell embryo, 4 cell embryo, 8 cell embryo and dividing morula (Figure 5 A, B, C, D and E) by IIF. No immunostaining of the cumulus cells was observed. The pre-immune serum serving as negative control did not show any immunoreactivity to any of the stages in embryogenesis or to the cumulus cells (represented in panel F). Monoclonal antibody to HSP90 on oocytes was used as positive control for the experiment (data not shown as it has been discussed in [12]).

## Discussion

Heat shock proteins are ubiquitous, phylogenetically conserved stress proteins which are known to have an important role in stress survival. Heat shock protein 90 is a hub in developmental regulatory networks. This protein plays a key role in various cellular processes as seen in the post translational maturation of proteins such as steroid hormone receptors, protein kinases and helix-loop-helix transcription factors [37]. Amongst all the molecular chaperones HSP90 is the most common one and apart from its chaperoning function in the cell, this dynamic molecule is involved in many cellular and pathophysiological processes such as maintenance of cytoarchitecture, differentiation and cytoprotection [38]. Approximately 1% of the total cellular proteome is constituted by this complex protein and it plays a critical role both in its normal function as a chaperone as well as its response to cellular stress. These unique properties of the protein render it to be of evolutionary significance and thus make it a well conserved protein right from prokaryotes. HSP90 comprises of 2 distinct isoforms: HSP90α and HSP90β which are encoded by 2 different genes at 14q 32-33 and 6p21 loci respectively. Though 85% similar, the isoform specificity is not only restricted to the biochemical level but it also extends to the functional role of HSP90 in cell differentiation and development [38]. Apart from its dogmatic role as a molecular chaperone, the isoform HSP90β is also known to be functionally important in reproduction. Using 2D proteomics and MS/MS, it was found that, among the hundreds of proteins that are expressed by the membrane of mammalian oocyte, HSP90 is one of the abundant proteins present on the oolemmal membrane of mouse eggs [39]. They demonstrated that HSP90 was primarily localized to the membrane' microvillar region of normal mouse eggs. Overexpression of HSP90β is observed throughout the germ-cell lineage from very early stages of development to adult oocytes and spermatocytes, thus establishing the important role of HSP90β in reproduction [40]. Studies have also shown that HSP90β isoform plays a major role in trophoblast differentiation and HSP90β -deficient homozygous mice fails to differentiate to form placental labyrinths [41].

In our previous report, we demonstrated the presence of anti-HSP90β antibodies in women with POI and repeated IVF-ET failure [12]. We verified the presence or absence of isoform specific HSP90 in ovarian extracts by Western Blot analysis and found that HSP90β is the predominant isoform present in the human ovarian extracts (Figure 1A). Having established this, we embarked on identifying, characterizing and validating the epitope/s of HSP90β using sera of infertile patients having these auto-antibodies.

The human HSP90β protein as documented in SwissProt is a 724 amino acids long constitutive cellular protein, which gets processed to a 723 amino acids long protein, by removal of its initiator methionine [42]. Based on the epitope prediction algorithms, 10 peptides of the beta isoform of HSP90 (labeled as EP1 to EP10) were shortlisted and were custom synthesized by solid-phase synthesis using Fmoc chemistry. The reactivity of these peptides with the sera of patients having HSP90 autoantibody and controls was checked and measured by ELISA. To determine the most immunodominant epitope we calculated the change in percent absorbance of all the 10 peptides and found that, EP6 demonstrated the highest change in percent absorbance followed by EP1 and EP8.

**Figure 6:**
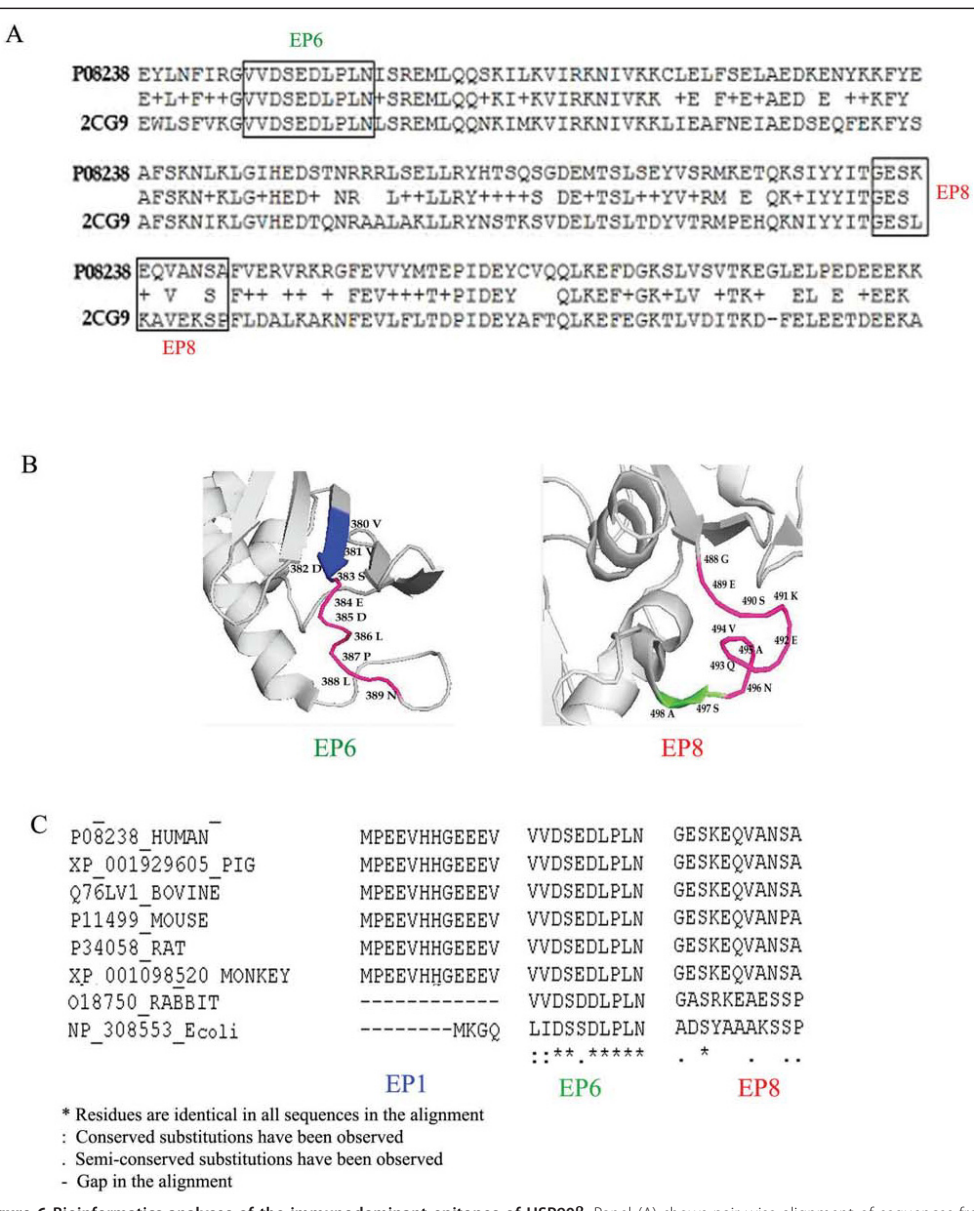
**Bioinformatics analyses of the immunodominant epitopes of HSP90β**. Panel (A) shows pair wise alignment of sequences from human HSP90β (SwissProt Accession No: P08238) and yeast HSP90 (PDB ID: 2CG9). The regions of the predicted epitopes in the alignment are highlighted in boxes. Panel (B) The structural coordinates are missing for the EP1 peptide. The structure of yeast HSP90 (PDB ID: 2CG9) has been used to represent the structures of peptides EP6 and EP8. The predicted antigenic regions are colored based on their secondary structures. Helices, strands and loops are colored green, blue and pink respectively. Panel (C) shows the conservation of the 3 putative dominant peptides across various species. It can be clearly seen that peptide EP6 is highly conserved antigen in all the species and therefore is one of the potential immune targets of HSP90β. The structures were rendered using the online algorithm PyMOL.

Structure analyses of these 3 immunodominant peptides reveal that they are surface exposed and hence accessible to the auto-antibody. The structure of the first 220 residues of human HSP90 has been experimentally elucidated [43]. Although peptide EP1 (1-12) lies in this region (Figure 1B), the structural coordinates are missing for the first 15 residues. In order to predict the structural features of EP6 and EP8, the protein structure database (PDB) was searched using the BLAST algorithm [44] which revealed that yeast HSP90 shared an identity of 62% and was the best template for predicting the structures of EP6 and EP8. The pairwise alignment of human HSP90β and yeast HSP90 showed that EP6 peptide shared 100% identity and EP8 peptide showed an identity match for 5 of its 11 residues (Figure 6A). EP6 was the most immunodominant antigenic decapeptide (VVDSEDLPLN) spanning the region 380-389 and is shown to be highly conserved across most species (Figure 6C); even its most distant relative, the HSP90 of *E.coli *exhibits sequence identity with 7 of the 10 residues of EP6 peptide. The four known important determinants of B-cell epitopes are surface accessibility, hydrophilicity, flexibility of the conformations and the presence of secondary structures such as beta-turns [45]. Cursory analysis of the sequences of peptides EP1, EP6 and EP8 reveal that they are abundant in hydrophilic and charged amino acids like Asp, Glu and Lys (Figure 1B). The 3D structures of peptides EP6 and EP8 reveal that the major fractions of their residues are present in the loop conformation (Figure 6B). Loops are the most mobile part of the protein structure. Since the complete structure of human HSP90 has not been experimentally elucidated, it is difficult to precisely comment on the surface accessibility of the residues in these peptides. However, since loop conformations are observed predominantly on the surface rather than the protein interior [46] and together with the highly polar and hydrophilic nature of these peptides, it can be safely predicted that these regions (EP1, EP6 and EP8) in the native protein will be accessible to the anti- HSP90 antibodies in circulation.

The presence of autoantibodies to HSP90 has been reported in the human natural antibody repertoire [47]. Thus, to rule out the false positive results arising from such a finding and to determine the cut off value for each of the 10 peptides, the ELISA data obtained was statistically analyzed and we quoted the two outer centiles such as the 80^th ^centile of controls and 20^th ^centile of patients to represent the data. Further, to determine the most immunodominant peptide, we calculated the change in percent absorbance of each of the peptide and found that of all the peptides, EP6 shows a significant increase in the percent change in absorbance between patients and controls. Thus, further validation and characterization were carried out using EP6 peptide. A polyclonal antibody to this peptide was raised and its reactivity was checked by Western Blot and immunofluorescence. It was found that the EP6 peptide polyclonal antibody not only recognizes the native protein in the mice ovarian extracts (Figure 4) but also exhibits similar immunostaining pattern with the oocyte and embryos (Figure 5) as was observed by a similar staining pattern by HSP90 autoantibody positive patient sera in our earlier study [12].

AOA are associated with poorer treatment outcomes in infertility patients. It has been shown by researchers that AOA could reduce fertilization rates, generate a poor response to gonadotropin stimulation, decrease pregnancy rates, affect egg and embryo development and could be responsible for implantation failures [48,49]. Knowing the AOA status in the infertile women and planning appropriate immunosuppressive therapy would not only ensure the effectiveness of the IVF-ET but also reduce the time invested by the clinician and the cost to be incurred by the patients and thereby restore fertility [50].

The precise mechanism of anti-HSP90 antibody related inhibition of embryo development and ovarian failure has not been reported. Recent reports have suggested that the penetration of autoantibodies into living cells participate in the pathogenesis of diverse autoimmune diseases. For instance, autoantibodies to HSP27, which are found in patients with glaucoma, have been shown to penetrate into human retinal neuronal cells and induce their active death, most likely by inactivating the ability of HSP27 to stabilize actin cytoskeleton thus suggesting a pathogenic role of these antibodies [51]. Also, there are increasing evidences to suggest the presence of HSP90 on the cell surface [39,52,53] thereby making it accessible to the autoantibodies. Thus, mere presence of these autoantibodies in the circulation may not only bring detrimental effects by evading the surface HSP90 but they can also get internalized into the cell and destroy the ovarian cytoarchitecture.

Till date there are no reports on the clinical effect of circulatory anti-HSP90 antibodies. A group demonstrated that inclusion of anti-HSP90 antibodies in cell culture medium exerted a detrimental effect on mouse embryo development and thus concluded that immune sensitization to heat shock proteins may be a cause of reproductive failure [54]. Studies have shown that HSP90β isoform plays a major role in trophoblast differentiation [41]. Also, in our earlier study we have demonstrated that patient's sera having autoantibody to HSP90 exhibits reactivity with the cells of developing embryo as well as the cells of the trophectoderm of the invading blastocyst which is the region known to form the placenta [12]. In the present study we confirmed the same observations using polyclonal antibodies to EP6 peptide. Thus, by virtue of its reactivity with oocytes and early developmental stages the mere presence of these autoantibodies would have detrimental effect on early stages of embryonic development and possibly leading to a pregnancy failure. However, the precise mechanism of anti-HSP90 antibody related inhibition of embryo development and ovarian failure has not been reported and needs to be investigated.

## Conclusions

The present study conclusively demonstrates that peptide EP6 of HSP90β is a major immunogenic epitope and thus is an ideal candidate marker for the protein. The ELISA clearly indicates that of all the 10 peptides, this is the only peptide which shows immunoreactivity with all of the 64 HSP90β positive patients' sera. The data also indicates that in addition to peptide EP6, other peptides such as EP1 and EP8 could also serve as potential epitopes. Identification and validation of protein epitopes can in the long run be of immense importance when quick, specific and sensitive immunodiagnostics are developed for screening patients with autoimmune diseases. Understanding of binding epitopes on the protein molecules is necessary to understand the pathophysiological events of a disease. The study might generate new tools for the detection of disease-inducing epitopes and a possible therapeutic intervention. Thus, it seems important to determine the epitopes of HSP90, such as those defined in this study, which are involved in responses of autoimmune diseases. Studies addressing these issues are of our importance and work towards this direction is underway.

## Competing interests

The authors declare that they have no competing interests.

## Authors' contributions

ESP and VVK designed the study and wrote the manuscript. ESP planned the experiments and with AKC, both conducted all the wet-lab experiments. SIT was responsible for the Bioinformatics analyses of the manuscript. All the four authors contributed towards the discussion and conclusions of the manuscript. All authors read and approved the final manuscript.
